# Glutamine metabolism-related genes predict prognosis and reshape tumor microenvironment immune characteristics in diffuse gliomas

**DOI:** 10.3389/fneur.2023.1104738

**Published:** 2023-03-10

**Authors:** Huanhuan Fan, Shuxin Zhang, Yunbo Yuan, Siliang Chen, Wenhao Li, Zhihao Wang, Yufan Xiang, Junhong Li, Xiaohong Ma, Yanhui Liu

**Affiliations:** ^1^Psychiatric Laboratory and Mental Health Center, West China Hospital of Sichuan University, Chengdu, Sichuan, China; ^2^West China Brain Research Center, West China Hospital of Sichuan University, Chengdu, Sichuan, China; ^3^Department of Neurosurgery, West China Hospital of Sichuan University, Chengdu, Sichuan, China; ^4^Department of Neurosurgery, Chengdu Second People's Hospital, Chengdu, Sichuan, China

**Keywords:** glutamine metabolism, diffuse glioma, tumor microenvironment, immune, prognosis

## Abstract

**Background:**

Diffuse gliomas possess a kind of malignant brain tumor with high mortality. Glutamine represents the most abundant and versatile amino acid in the body. Glutamine not only plays an important role in cell metabolism but also involves in cell survival and malignancies progression. Recent studies indicate that glutamine could also affect the metabolism of immune cells in the tumor microenvironment (TME).

**Materials and methods:**

The transcriptome data and clinicopathological information of patients with glioma were acquired from TCGA, CGGA, and West China Hospital (WCH). The glutamine metabolism-related genes (GMRGs) were retrieved from the Molecular Signature Database. Consensus clustering analysis was used to discover expression patterns of GMRGs, and glutamine metabolism risk scores (GMRSs) were established to model tumor aggressiveness-related GMRG expression signature. ESTIMATE and CIBERSORTx were applied to depict the TME immune landscape. The tumor immunological phenotype analysis and TIDE were utilized for predicting the therapeutic response of immunotherapy.

**Results:**

A total of 106 GMRGs were retrieved. Two distinct clusters were established by consensus clustering analysis, which showed a close association with the IDH mutational status of gliomas. In both IDH-mutant and IDH-wildtype gliomas, cluster 2 had significantly shorter overall survival compared with cluster 1, and the differentially expressed genes between the two clusters enriched in pathways related to malignant transformation as well as immunity. *In silico* TME analysis of the two IDH subtypes revealed not only significantly different immune cell infiltrations and immune phenotypes between the GMRG expression clusters but also different predicted responses to immunotherapy. After the screening, a total of 10 GMRGs were selected to build the GMRS. Survival analysis demonstrated the independent prognostic role of GMRS. Prognostic nomograms were established to predict 1-, 2-, and 3-year survival rates in the four cohorts.

**Conclusion:**

Different subtypes of glutamine metabolism could affect the aggressiveness and TME immune features of diffuse glioma, despite their IDH mutational status. The expression signature of GMRGs could not only predict the outcome of patients with glioma but also be combined into an accurate prognostic nomogram.

## Introduction

Diffuse gliomas are thought to arise from glial or glial precursor cells, possess remarkable heterogeneous characteristics, and represent a kind of malignant brain tumor with high mortality (1, 2). Diffuse gliomas are categorized with a range of World Health Organization (WHO) grades 2, 3, and 4, and their histological types include astrocytoma, oligodendroglioma, and mixed glioma. Unlike WHO grade 1 circumscribed gliomas, which are potentially curable by complete surgical resection, diffuse gliomas are almost incurable due to their infiltrative features (3). Although surgical techniques and anti-cancer drugs have progressed, there exist little and even no changes in the dismal prognosis and treatment options for diffuse gliomas (4). Since the fourth edition of the WHO's classification of tumors was updated in 2016, molecular biomarkers were first introduced in the diagnosis and classification of diffuse glioma, which opened a new era for glioma research.

Glutamine is an L-α-amino acid and is nutritionally classified as a non-essential amino acid, which represents the most abundant and versatile amino acid in the body (5). Glutamine plays an important role in cell metabolism, including participating in the tricarboxylic acid (TCA) cycle and the biosynthesis of nucleotides, redox balance, and other non-essential amino acids (6, 7). Glutamine is also a crucial nutrient required for the survival and progression of various malignancies. Numerous types of cancers are characterized by increased glutamine consumption mainly by regulating the activity of glutamine-related enzymes such as glutamine synthetase (GS) and glutaminase (GLS) (8). Therefore, glutamine metabolism-related specific pathways have been ideal targets for developing anti-cancer drugs, with glutamine uptake inhibitors, glutamine antimetabolites, and glutaminase inhibitors being potential approaches (9, 10). In Jin et al.'s (11) study, a single glutaminase inhibitor had limited the anti-cancer effect, and the dual inhibition of glutamine metabolism by targeting both glutaminase and glutamine transporter showed a promising therapeutic effect. Nowadays, patient stratification and drug combination strategies have been applied to maximize the efficacy of glutamine metabolism inhibitors as tumor therapeutics (9).

Glutamine could also affect the metabolism of immune cells in the tumor microenvironment (TME), which indirectly leads to the appearance of a distinct immune landscape. Glutamine is utilized at a high rate by immune cells and is necessary for supporting lymphocyte proliferation and the production of cytokines by lymphocytes and macrophages (12). An inverse relationship between glutamine metabolism and T cell cytotoxicity and worse prognosis was observed in patients with breast cancer whose tumors harbor both high glutamine metabolism and low T cell cytotoxicity signatures (13). In addition, growing evidence indicates that glutamine enhances the function of immune cells including B lymphocyte differentiation, antigen presentation, and macrophage phagocytosis (14). A previous study discovered that targeting glutamine metabolism inhibited the generation and recruitment of myeloid-derived suppressor cells (MDSCs) and promotes the generation of antitumor inflammatory tumor-associated macrophages (TAMs) (15), while a specific loss of GLS could improve antitumor T cell activation (13).

Tumors that arise in different organs can have distinct phenotypes for glutamine metabolism (7). In the current study, we made an attempt to clarify the relationship between glutamine metabolism and diffuse glioma and figure out whether glutamine metabolism affected the progression of diffuse glioma and reshaped the TME features based on the amount of transcriptome data.

## Materials and methods

### Data screening and retrieve

The transcriptome data and corresponding clinicopathological information of patients with glioma were acquired from the Cancer Genome Atlas (TCGA, https://www.cancer.gov/) (16) and Chinese Glioma Genome Atlas (CGGA, http://www.cgga.org.cn/) (17), respectively. In total, one cohort from TCGA and two cohorts (CGGA325 and CGGA693) from CGGA were collected. A patient cohort from West China Hospital (WCH) was also included in the current research. After surgery, the patients were followed up every 3–6 months for assessing the prognosis. Overall survival (OS) was defined as the duration from the date of operation to death or the end of the observation period. Exclusion criteria include those as follows: (1) younger than 18 years old, (2) dead or censored within 30 days after surgery, (3) incomplete clinicopathological data, and (4) recurrent gliomas.

Clinicopathological characteristics of patients with diffuse glioma from four cohorts are listed in Table 1.

**Table 1:** Clinicopathological characteristics of patients with diffuse glioma in the TCGA, CCGA, and WCH cohort.

**Clinicopathological variables**	**TCGA**	**CGGA325**	**CGGA693**	**WCH**
Sample size	622	218	396	77
Normal brain	5	20	20	16
Age	46 (18–87)	43 (18–79)	43 (18–76)	46 (19–81)
**Gender**				
Female	284	115	278	32
Male	369	189	368	51
**Histology**				
Astrocytoma	335	108	272	24
Oligodendroglioma	158	60	141	21
Glioblastoma	160	132	233	38
NA	0	4	0	0
**WHO grade**				
G2	206	97	168	31
G3	228	71	245	14
G4	160	132	233	38
NA	59	4	0	0
**IDH status**				
WT	229	139	267	45
Mutant	412	164	331	38
NA	12	1	48	0
**1p19q codeletion status**				
Non-codel	481	234	443	46
Codel	158	62	137	19
NA	14	8	66	18
TERT promoter status		–	–	
WT	157	–	–	32
Mutant	330	–	–	27
NA	166	–	–	24
**MGMT promoter status**				
Unmethylated	149	138	210	16
Methylated	463	148	301	38
NA	41	18	135	29
ATRX status		–	–	
WT	441	–	–	25
Mutant	193	–	–	55
NA	19	–	–	3

TCGA, the cancer genome atlas; CGGA, Chinese glioma genome atlas; WCH, west China hospital; WHO, World Health Organization; IDH, isocitrate dehydrogenase; codel, codeletion; TERT, telomerase reverse transcription; MGMT, O6-methylguanine-DNA methyltransferase; ATRX, alpha thalassemia/mental retardation syndrome X-linked protein/gene; WT, wild; NA, not available.

### RNA disposal and analysis

Frozen diffuse glioma and adjacent normal brain tissue specimens from West China Hospital were homogenized, and TRIzol reagent (Invitrogen, USA) was utilized to isolate the total RNAs. After quality checking, 2 μg RNA per sample was used as the input material for the RNA sample preparations. mRNA was purified from the total RNAs by using poly-T oligo-attached magnetic beads. Then PCR products were purified by the AMPure XP system. mRNA was reverse-transcribed into a cDNA library and was sequenced on the Illumina NovaSeq S6000 platform to generate 150 bp paired-end reads. Clean reads were mapped to the hg19 genome and counted using STAR (2.6.0c).

### Glutamine metabolism-related gene (GMRG) screening

The GMRGs were retrieved from the Molecular Signature Database (MSigDB, https://www.gsea-msigdb.org/) (18) using the R package msigdbr, which included the following pathways: glutamine family amino acid metabolic process, glutamine family amino acid biosynthetic process, glutamine family amino acid catabolic process, glutamine metabolic process, glutamate and glutamine metabolism, carbon–nitrogen ligase activity with glutamine as amido *N* donor, glutamine transport, protein-glutamine gamma-glutamyltransferase activity, L glutamine transmembrane transporter activity, regulation of glutamine family amino acid metabolic process, and peptidyl glutamine methylation (Supplementary Table S1). Genes were excluded if they were not present in the RNA expression data of all four datasets or if their expressions were too low (maximum FPKM < 0.1) in the TCGA RNA-seq data.

### Unsupervised consensus clustering

Consensus clustering analysis was used to explore expression patterns of GMRGs. Briefly, for *k* numbers from 2 to 10, the hierarchical clustering of *k* clusters based on Pearson correlation was performed over 1,000 random subsets of samples, and the frequency that two samples were clustered in the same group was calculated as the consensus index. The optimal *k* was determined if the cumulated distribution function (CDF) of the consensus index approximated a zigzag shape, and the sample size of each cluster was not too small to study its implications. After consensus clustering analysis on the TCGA dataset, we trained a random forest classifier using the TCGA dataset to classify samples in other cohorts into the consensus clusters.

### Mutation analysis

To understand the association between GMRG expression and genetic alterations of gliomas, we downloaded somatic mutation calls of patients in the TCGA cohort curated by the cBioPortal database (https://www.cbioportal.org/). The “oncoplot” package from R package “maftools” was utilized to visualize the gene alterations and annotate GMRG clustering results as well as other clinical information.

### Glutamine metabolism-related risk score construction and validation

Patients with diffuse glioma from TCGA were randomly divided into a training group and a validation set with a ratio of 6:4. In the training group, glutamine metabolism-related risk score (GMRS) was performed to establish a prognostic predictive method based on GMRGs and patients with diffuse glioma. Using the “glmnet” R package, the univariate Cox regression and the least absolute shrinkage and selection operator (LASSO) Cox regression analysis were used to screen for the strongest prognostic signature while minimizing the risk of over-fitting. One thousand LASSO Cox regression models were generated using different random number seeds. GMRGs with non-zero coefficients in over 700 models were selected, and the GMRS was calculated with the following algorithm:


$$\begin{array}{l} GMRS=\;\underset{i=1}{\sum }\beta_{i}^{*}Exp_{i}. \end{array}$$

In the formula, *Exp* represents the expression level and β represents the coefficient of each prognostic GMRG in the final Cox regression model. An optimal cutoff value for GMRS to divide the specific cohort into two risk groups was determined using the “survminer” R package. For the TCGA dataset, the cutoff was derived from the training set and evaluated in the test set. The cutoffs for other datasets were calculated based on their own GMRS distribution due to differences in the generation process of the transcriptome data. The group with GMRS value < optimal cutoff value was defined as a low-risk group, while GMRS value ≥optimal cutoff value was defined as a high-risk group.

At first, the optimal value of GMRS was calculated in the training group. Then, the calculated optimal cutoff value was applied in the validation group, and the prognostic ability of GMRS was verified. Patients from CGGA and WCH were used as the external group for further validation. Each cohort possessed a unique optimal cutoff value base on the algorithm. Kaplan–Meier (K–M) curves and time-dependent receiver operating characteristic (ROC) curves of the GMRS at 1, 2, and 3 years were depicted.

### Construction and validation of a nomogram for predicting prognosis

To determine the independent prognostic role of GMRS, we conducted univariate and multivariate Cox regression analyses based on variables including the GMRS, age, sex, WHO grade, Karnofsky Performance Scale (KPS), isocitrate dehydrogenase (IDH) mutational status, 1p/19q codeletion status, chemotherapy, and radiotherapy. Variables with a *p*-value of < 0.1 in univariate Cox analysis were included in multivariate Cox regression for further analysis. Variables with a *p*-value of < 0.05 in multivariate Cox regression analysis were regarded as independent risk factors. A prognostic nomogram was conducted based on the independent risk factors using the “rms” R package. The efficacy of the nomogram was assessed by the calibration curve. Nomograms were formed and assessed in the four cohorts.

### Functional enrichment analysis of DEGs

Differentially expression genes between different groups (cluster 1 vs. cluster 2) with an adjusted *p*-value below 0.05, and log_2_ (fold change) >0.5 or below −0.5 were defined as GMRS-associated DEGs. Gene-set enrichment analysis (GSEA) of DEGs was performed and visualized by using the “clusterProfiler” R package. Gene set variation analysis (GSVA) was conducted using the “GSVA” R package. The differential expression of gene sets was conducted using limma. The Kyoto Encyclopedia of Genes and Genomes (KEGG), Reactome, Hallmark, and Gene Ontology: Biological Process (GO: BP) gene sets from MSigDB were used as the gene set database for the functional analysis.

### Immune landscape depiction

Estimation of stromal and immune cells in malignant tumor tissues using expression data (ESTIMATE, https://bioinformatics.mdanderson.org/) is a tool to predict tumor purity and the presence of infiltrating stromal/immune cells based on gene expression data (19). The stromal score (the presence of stroma in the tumor tissue), immune score (the infiltration of immune cells in the tumor tissue), and estimate score were calculated through the ESTIMATE algorithm. Tumor purity was estimated as described by K Yoshihara et al. based on the ESTIMATE results. CIBERSORTx (https://cibersortx.stanford.edu/) provides an estimation of the abundance of member cell types in a mixed cell population by using gene expression data (20). Immune Cell Abundance Identifier (ImmuCellAI) is also a useful tool to assess the abundance of immune cells from the gene expression dataset including RNA-seq and microarray data (21).

Correlations between glutamine metabolism clusters or risk groups and expression of immune checkpoints (ICPs) were analyzed.

### Prediction of therapeutic response by immunotherapy

On the basis of the extent of immune cell infiltration, tumors are now divided into “cold” (non-inflammatory) and “hot” (pro-inflammatory) types (22). “Hot” tumors are characterized by significant T cell infiltration and associated with better efficacy of immunotherapy (23). We distinguished diffuse gliomas into “cold” and “hot” immune phenotypes based on the methods described by Wang et al. (22).

Tumor immune dysfunction and exclusion (TIDE, http://tide.dfci.harvard.edu/) was utilized to predict the therapeutic response of immune checkpoint blockades (ICBs) in patients with diffuse glioma by evaluating the function of cytotoxic T lymphocytes (24).

### Statistical analysis

R software (version 3.6.1) and the above-mentioned package were used to handle the transcriptome data. Differences between glutamine metabolism subtypes and clinic variables were analyzed using Student's *t*-test or the chi-square test. K–M analysis was conducted using the log-rank test. A two-sided *p*-value of < 0.05 was regarded as statistically significant and ^*^ indicated a *p*-value of < 0.05, whereas ^**^ indicated a *p*-value of < 0.01, ^***^ a *p*-value of < 0.001, and ^****^ a *p*-value of < 0.0001 in the current study.

### Ethics statement

The Ethical Committee of West China Hospital Sichuan University approved this study (Ethic number: 2018.569), and all behaviors were conducted according to the principles expressed in the Declaration of Helsinki. Before surgery, all patients and their authorized trustees were informed and signed informed consent to use their clinical data for research purposes.

## Results

### Identification of glutamine metabolism subtypes in diffuse gliomas

**Figure 1:**
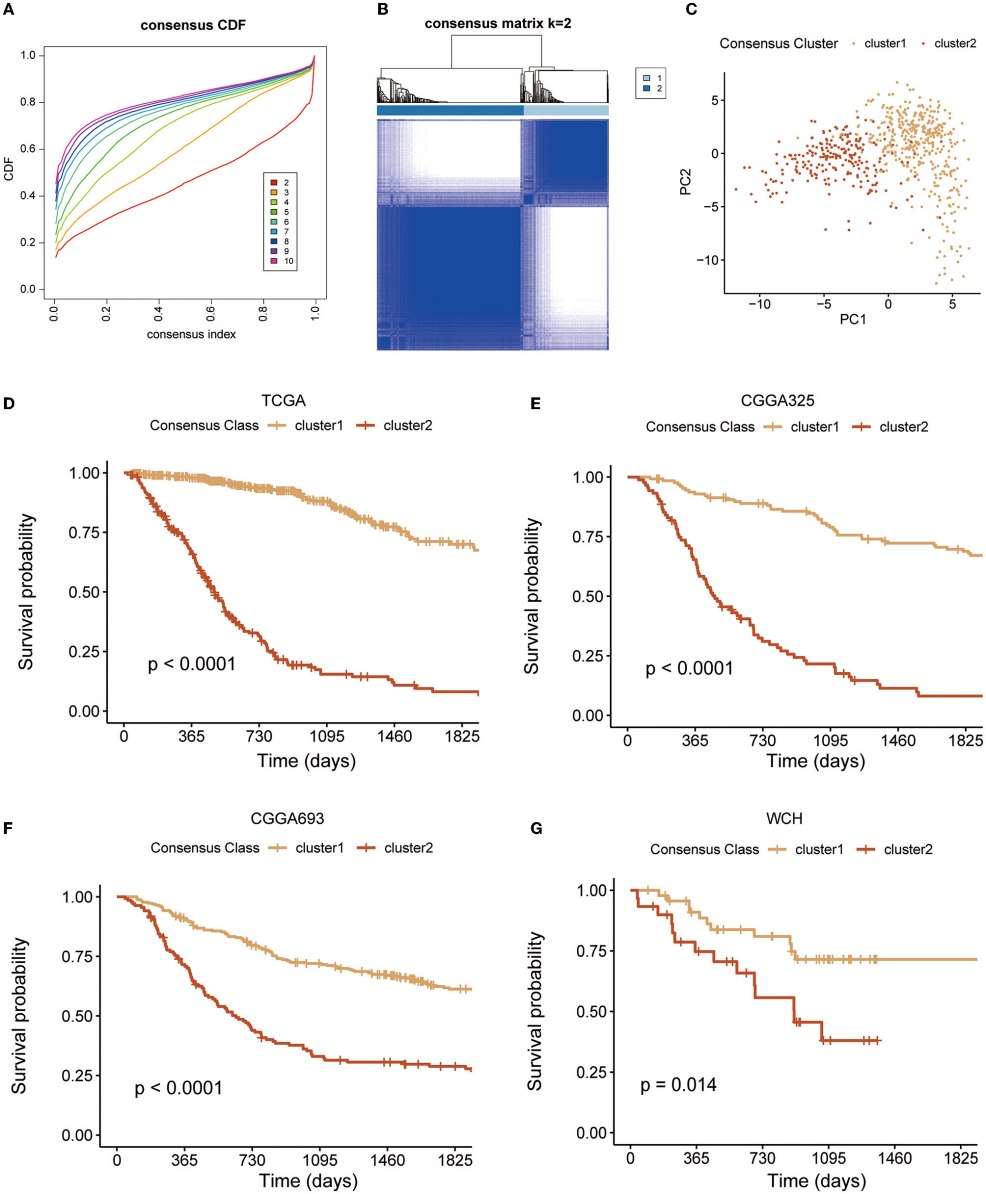
Unsupervised cluster analysis. **(A, B)** The clustering implying the optimal κ was 2; **(C)** two distinct clusters formation under the PCA algorithm; and **(D–G)** K–M curves of clusters in four diffuse glioma cohorts. PCA, principal component analysis; K–M, Kaplan–Meier.

A total of 106 GMRGs were retrieved from the MSigDB (Supplementary Table S1) and 87 GMRGs were expressed in all four cohorts. To determine the relationship between expression patterns of GMRGs and diffuse glioma, unsupervised consensus clustering analysis was conducted to classify patients with diffuse glioma from TCGA based on the expression levels of the GMRGs. According to the principles described in the section Methods, we found that all the CDF approximated a *Z* shape, but the sample sizes of the clusters were maximized when the number of clusters (*k*) equals 2. Therefore, the optimal k was determined to be 2 (Figures 1A, B), which means the entire TCGA cohort was divided into two clusters, i.e., cluster 1 (*n* = 394) and cluster 2 (*n* = 228) (Figure 1C).

**Figure 2:**
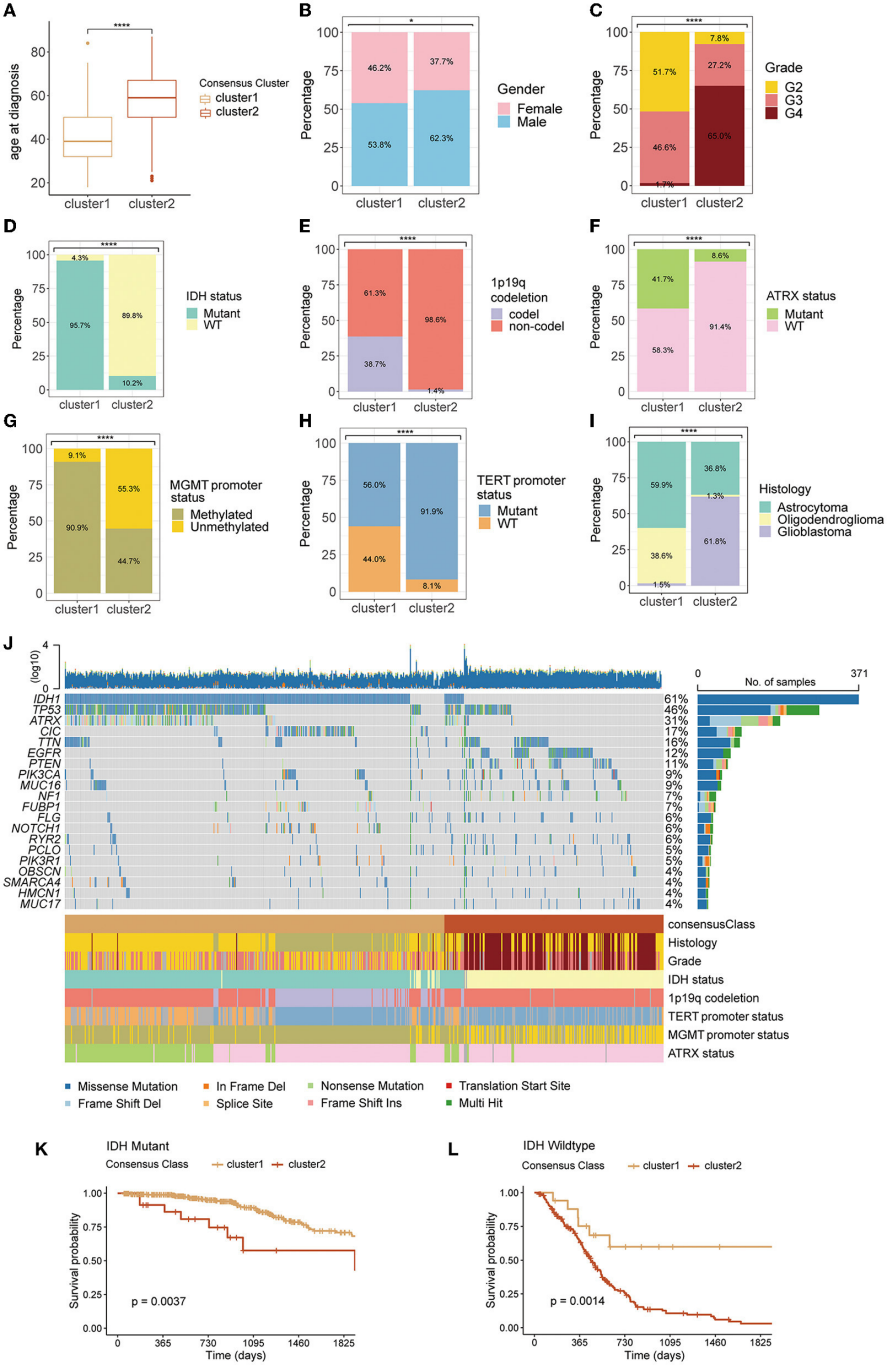
Differences in clinicopathological variables between clusters 1 and 2: age at diagnosis **(A)**, gender **(B)**, WHO grade **(C)**, IDH mutation status **(D)**, 1p19q codeletion status **(E)**, ATRX mutation status **(F)**, MGMT promoter status **(G)**, TERT promoter status **(H)**, and histology **(I)**, the waterfall plot depicting the top 20 mutant genes in the entire TCGA cohort **(J)**, difference between clusters 1 and 2 in overall survival for patients with IDH-mutant **(K)**, or IDH-wildtype gliomas **(L)**. WHO, World Health Organization; IDH, isocitrate dehydrogenase; codel, codeletion; TERT, telomerase reverse transcription; MGMT, O6-methylguanine-DNA methyltransferase; ATRX, alpha thalassemia/mental retardation syndrome X-linked protein/gene; TCGA, The Cancer Genome Atlas.

The pattern of clustering in TCGA was also applied in other cohorts. The K–M curves (Figures 1D–G) indicated that cluster 1 had a significantly longer OS compared with cluster 2 in four cohorts. As shown in Figures 2A–I, there existed a significant difference between clinicopathological features and clusters. The waterfall plot (Figure 2J) displayed the top 20 mutation genes in the entire TCGA cohort. The common mutation genes included *IDH1, TP53, ATRX, CIC, TIN, EGFR*, and *PTEN*. Notably, the GMRG expression clustering of gliomas was significantly associated with the mutational status of IDH genes (Figures 2D, J), which has been a known determinant for the survival outcome of patients with glioma. To further elucidate the prognostic value of GMRG expression clusters in this context, we split the TCGA cohort according to the IDH mutational status and found that the cluster 1 gliomas had a significantly better outcome compared to cluster 2 gliomas in both IDH-mutant and IDH-wildtype gliomas (Figures 2K, L).

**Figure 3:**
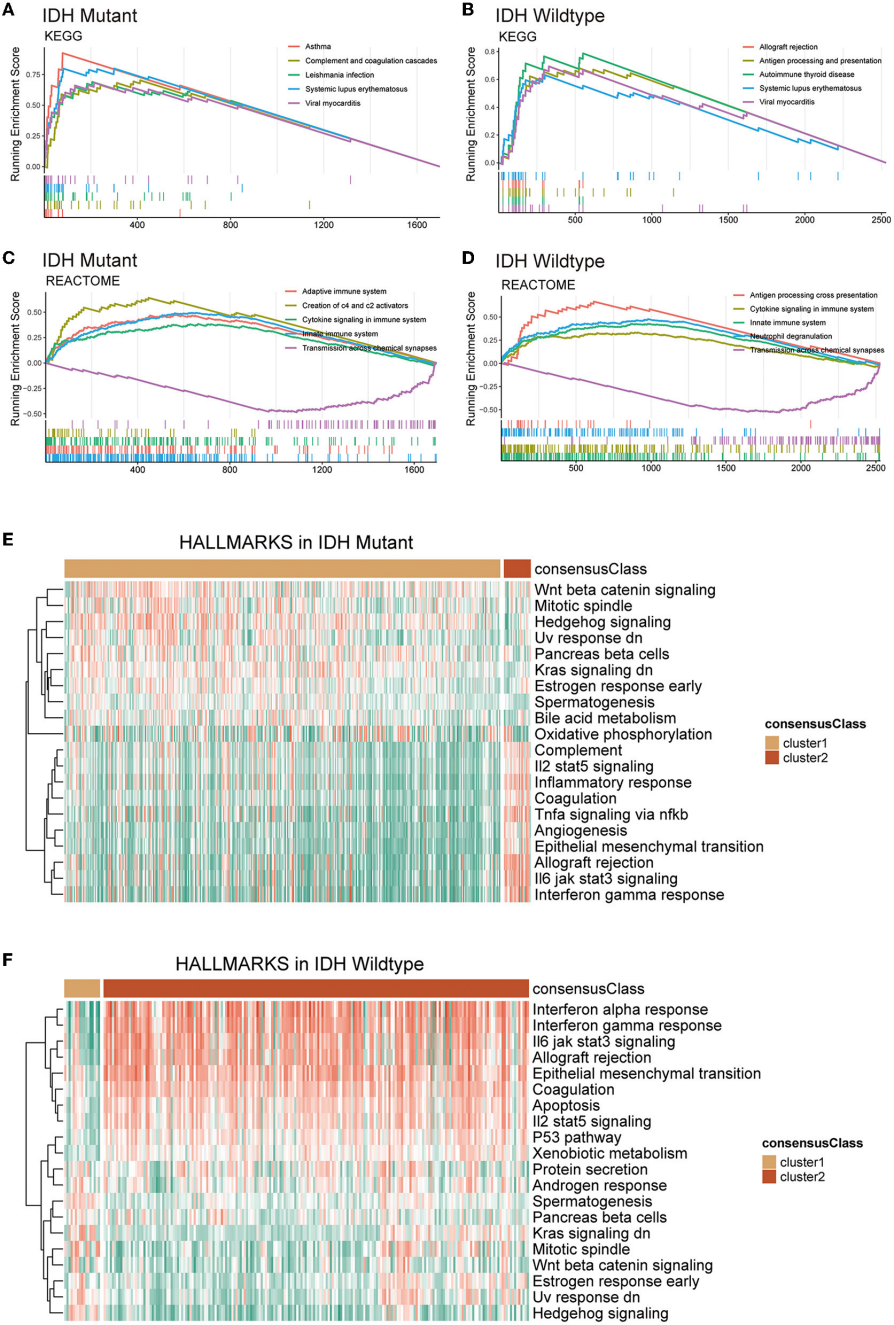
Functional enrichment analysis of DEGs between clusters 1 and 2 based on IDH mutation status. Top 5 KEGG pathways **(A, B)** and reactome pathways **(C, D)** based on GSEA. The heatmap of GSVA based on Hallmark gene sets in IDH-mutant **(E)** and IDH-wildtype **(F)** gliomas. DEG, differentially expression gene; IDH, isocitrate dehydrogenase; KEGG, Kyoto Encyclopedia of Genes and Genomes; GSVA, gene set variation analysis; GSEA, gene set enrichment analysis.

Next, we conducted GSEA and GSVA to further reveal the underlying differences between two GMRG expression patterns with respect to the functional status of biological pathways. The GSEA results showed that in both IDH-mutant and IDH-wildtype gliomas, systemic lupus erythematosus, viral myocarditis, cytokine signaling in the immune system, innate immune system, and transmission across chemical synapses were among the top five enriched KEGG and Reactome pathways associated with the DEGs between clusters 1 and 2 (Figures 3A–D). In addition, the GSVA revealed that epithelial-mesenchymal transition, IL6-JAK-STAT3 signaling, IL2-STAT5 signaling, interferon-gamma response, allograft rejection, and coagulation Hallmark gene sets were expressed at a higher level in cluster 2 gliomas of both IDH subtypes (Figures 3E, F).

### Immune profiles of glutamine metabolism subtypes

**Figure 4:**
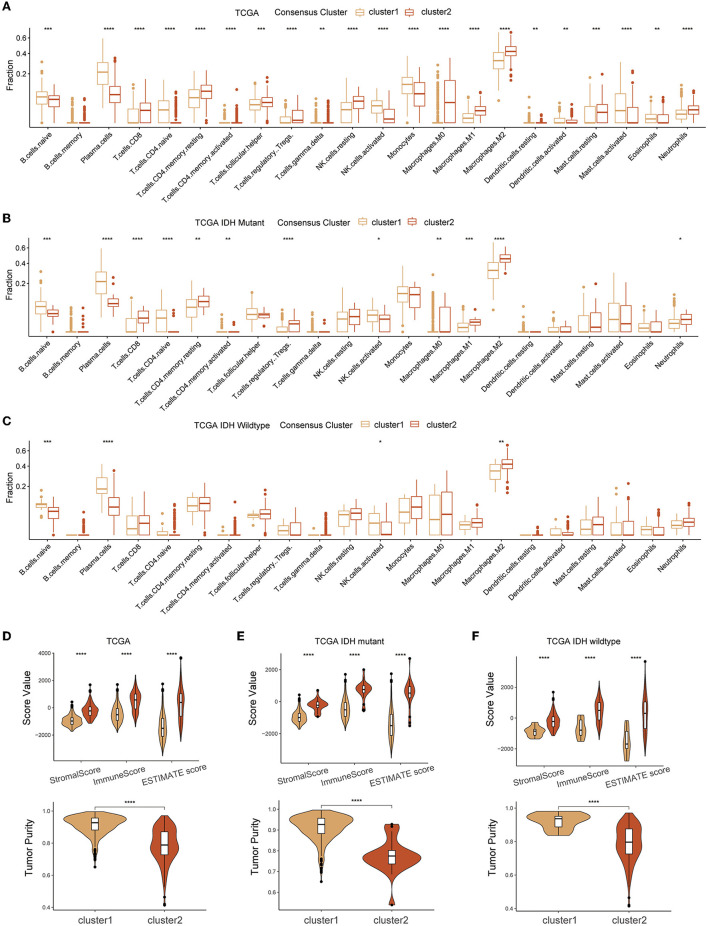
Association between TME infiltration of diffuse glioma and GMRG expression clusters. CIBERSORTx **(A–C)** and ESTIMATE **(D–F)** algorithm for evaluating TME immune characteristics of diffuse glioma in the entire TCGA cohort and subgroups based on IDH mutation status. TME, tumor microenvironment; GMRG, glutamine metabolism-related genes; ESTIMATE, estimation of stromal and immune cells in malignant tumor tissues using expression data; TCGA, The Cancer Genome Atlas; IDH, isocitrate dehydrogenase.

CIBERSORT and ESTIMATE algorithms were employed for exploring the TME immune characteristics in two clusters. Based on the results of the CIBERSORT algorithm, a substantial enrichment difference of the 22 immune cells between the two clusters was displayed (Figure 4A). The proportion of B cells, most T cells, natural killer (NK) cells, macrophages, eosinophils, neutrophils, etc., evidently showed a significant difference in enrichment levels. Of note, most types of T cells showed a comparatively high extent of infiltration in cluster 2. This result was partly consistent with the results of the other three cohorts (Supplementary Figures S1A–C). However, when the gliomas were split by IDH mutational status, the difference in T cells was not significant in IDH-wildtype gliomas, while the infiltration of naïve B cell and plasma cells was significantly lower in cluster 2 of both IDH subtypes, and M2 macrophages were more abundant in cluster 2 gliomas (Figures 4B, C). ESTIMATE analysis indicated that cluster 2 had a higher stromal score, immune score, estimate score, and lower tumor purity compared with cluster 1 in the different glioma cohorts and both IDH subtypes (Figures 4D–F and Supplementary Figures S1D–L).

**Figure 5:**
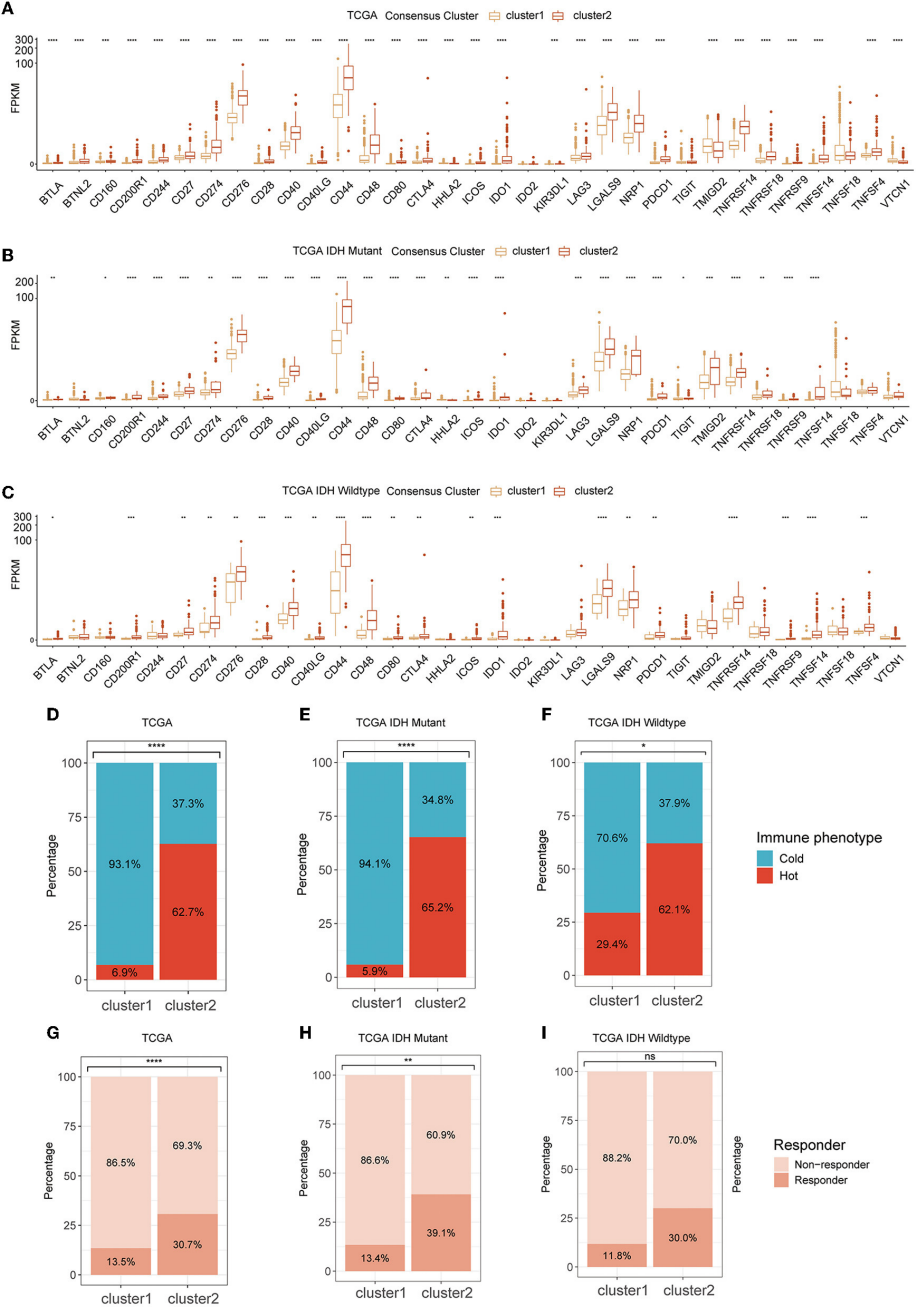
The expression level of ICPs in two clusters in TCGA cohort **(A)** and subgroups based on IDH mutational status **(B, C)**; tumor immunologic phenotype analysis for classified tumors as “cold” and “hot” **(D–F)**; and TIDE algorithm for predicting the therapeutic response **(G–I)**. ICP, immune checkpoint; TCGA, The Cancer Genome Atlas; IDH, isocitrate dehydrogenase; TIDE, tumor immune dysfunction and exclusion.

The RNA expression of immune checkpoints was also investigated for association with GMRG expression clustering. We found that cluster 2 had a remarkably high expression level of most of the immune checkpoints including PD-1 (PDCD1), PD-L1 (CD274), and CTLA-4 (CD276), and most of these trends were consistent between IDH-mutant and IDH-wildtype gliomas (Figures 5A–C and Supplementary Figures S2A–C).

As for the tumor immunological phenotype, the results indicated that cluster 2 possessed higher proportions of “hot” tumor phenotypes (Figures 5D–F and Supplementary Figures S2D–L). The therapeutic response of immunotherapy by ICBs was predicted by TIDE. The results indicated that cluster 2 had a larger population of patients who were more sensitive to immunotherapy (Figure 5G). In IDH-mutant and wildtype gliomas, although these differences were preserved in both subtypes, their significance was diminished possibly due to lower sample size (Figures 5H, I and Supplementary Figures S3A–I).

### Prognostic glutamine metabolism risk score development and validation

**Figure 6:**
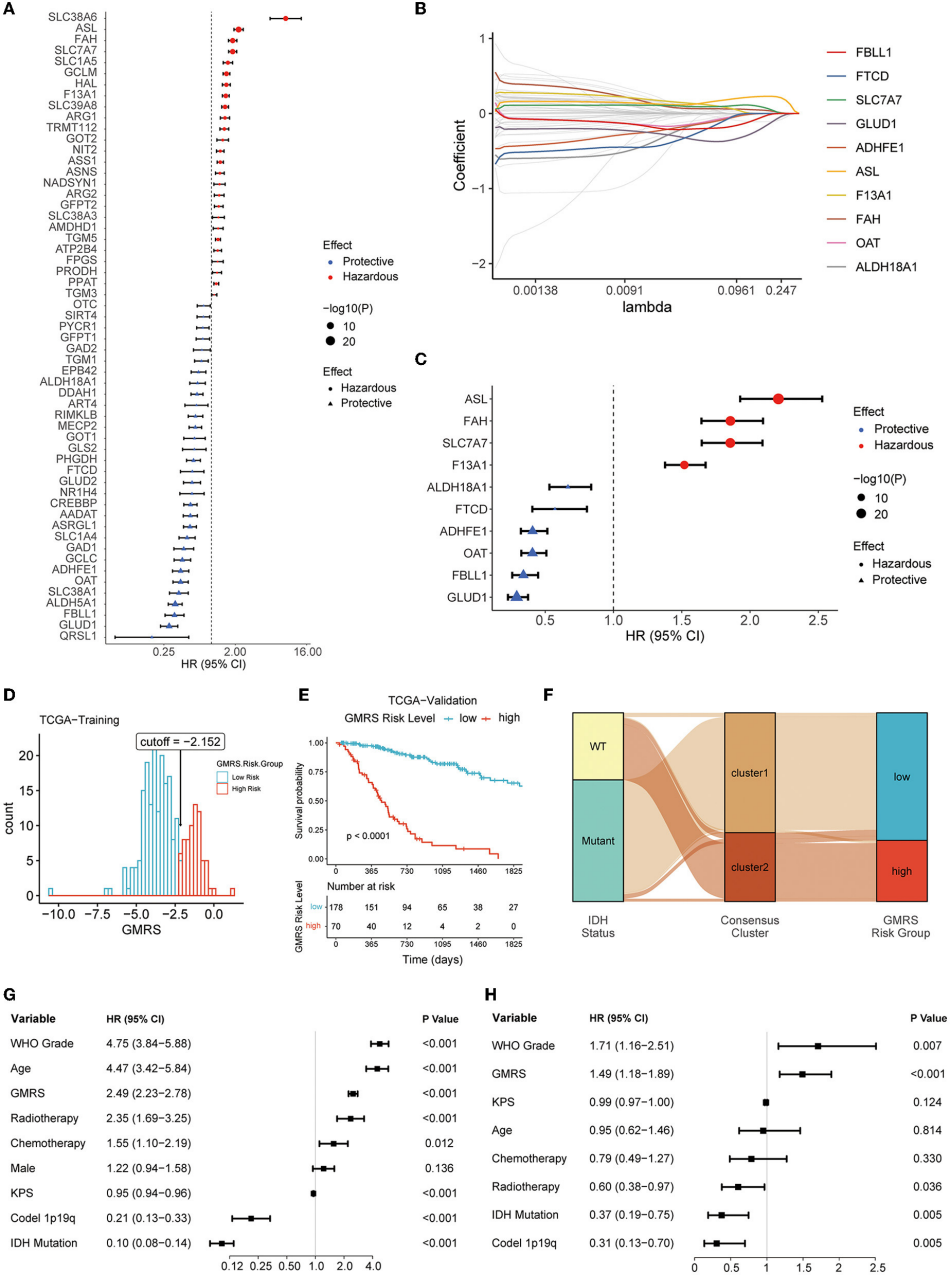
Screening of prognostic GMRGs. **(A)** Forest plot for preliminary screening prognostic GMRGs; **(B)** LASSO Cox regression for screening stable prognostic GMRGs; **(C)** forest plot for displaying 10 robust prognostic GMRGs; **(D)** optimal cutoff value of GMRS in the TCGA training group; **(E)** K–M curves for assessing GMRS in TCGA-validation group; **(F)** alluvial plot for showing the connection between clusters and GMRS risk groups; **(G)** univariate Cox regression of clinical variables in the entire TCGA cohort; and **(H)** multivariate Cox regression of clinical variables with a *p*-value of < 0.1 in univariate Cox regression in the entire TCGA cohort. GMRG, glutamine metabolism-related gene; LASSO, least absolute shrinkage and selection operator; GMRS, glutamine metabolism risk score; TCGA, The Cancer Genome Atlas; CGGA, Chinese Glioma Genome Atlas; WCH, West China Hospital.

First, in the TCGA training cohort, we conducted a univariate Cox regression analysis to screen the GMRGs. In the TCGA training set, 57 candidate GMRGs were remarkably associated with OS (Figure 6A). Then these candidate genes were filtered using repeated LASSO regression (Figure 6B). A total of 10 GMRGs (Figure 6C) were finally selected to build the glutamine metabolism risk score (GMRS). Among the 10 prognostic LMRGs, six predicted a better prognosis, while four predicted a worse prognosis.

The GMRS was calculated based on the algorithm as follows: 0.065 ^*^ ASL + 0.032 ^*^ SLC7A7 + 0.006 ^*^ F13A1 – 0.007 ^*^ FAH – 0.008 ^*^ GLUD1 – 0.012 ^*^ OAT – 0.044 ^*^ FBLL1 – 0.049 ^*^ ALDH18A1 – 0.056 ^*^ ADHFE1 – 0.972 ^*^ FTCD. The relationship between GMRS and clinicopathological characteristics of diffuse glioma was explored. The results showed that GMRS was significantly related to WHO grade, IDH mutation status, ATRX mutation status, MGMT promoter methylated status, TERT promoter mutation status, 1p19q codeletion status, and histology, but not sex (Supplementary Figures S4A–I). The impact of a single GMRG on clinic variables is depicted in Supplementary Figure S4J.

After calculating the optimal cutoff (optimal cutoff −2.152, Figure 6D) of GMRS, the TCGA training group was divided into a high-risk group and a low-risk group. The optimal cutoff value was also applied to the validation group. Based on the same algorithm, the prognostic capability of GMRS was also evaluated in CGGA and WCH cohorts (optimal cutoff −4.278 in CGGA325, −6.120 in CGGA693, and −0.549 in WCH) (Supplementary Figures S5A–C). The K–M analysis revealed that significant differences in OS were presented in all cohorts (Figure 6E and Supplementary Figures S5D–F), and the alluvial plot showed a close connection between cluster 1 and low GMRS risk gliomas, as well as between cluster 2 and high GMRS gliomas (Figure 6F).

To understand if the GMRS was an independent prognostic factor for gliomas, we conducted univariate Cox regression analysis to first identify other potential prognostic factors of gliomas in addition to GMRS (Figure 6G). Then, we evaluated the independence of GMRS using a multivariate analysis of GMRS and these factors (Figure 6H). Together, the evidence of the above-indicated GMRS was a robust prognostic predictor for patients with diffuse glioma.

### Nomogram construction based upon GMRS

**Figure 7:**
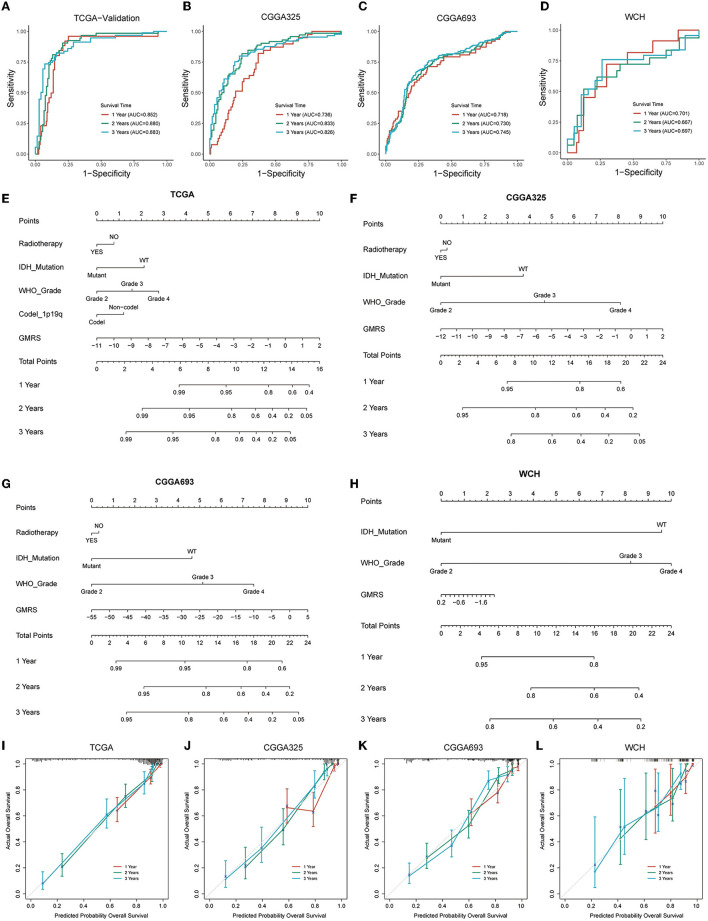
Prognostic predictive performance of GMRS and nomogram construction. **(A–D)** ROC curves for predicting 1-, 2-, and 3-year overall survival in the TCGA, CGGA325, CGGA693, and WCH cohort; **(E–H)** nomogram construction based on independent prognostic clinical variables; and **(I–L)** calibration curve for assessing the performance of each nomogram. GMRS, glutamine metabolism risk score; ROC, receiver operator characteristic; TCGA, The Cancer Genome Atlas; CGGA, Chinese Glioma Genome Atlas; WCH, West China Hospital.

To evaluate the performance of GMRS in estimating the aggressiveness of gliomas, ROC curves were applied for evaluating the predictive ability of GMRS in four cohorts. In the TCGA-validation group (Figure 7A), the AUC values of 1-, 2-, and 3-year survival were 0.852, 0.880, and 0.883, respectively. The AUC values of 1-, 2-, and 3-year survival were 0.736, 0.833, and 0.826 in the CGGA325 cohort (Figure 7B); 0.718, 0.730, and 0.745 in the CGGA693 cohort (Figure 7C); and 0.701, 0.667, and 0.697 in the WCH cohort (Figure 7D).

Next, to determine if the combination of GMRS and other independent prognostic variables could be more accurate in predicting the survival risks for patients with diffuse glioma, prognostic nomograms were established to predict 1-, 2-, and 3-year survival rates in the four cohorts (Figures 7E–H). Compared with using GMRS alone, the multivariate nomogram showed superiority in predicting the prognosis (C-index 0.849 vs. 0.829 in TCGA, 0.759 vs. 0.716 in CGGA325, 0.787 vs. 0.684 in CGGA693, and 0.685 vs. 0.630 in WCH). The calibration curves for verifying the efficacy of nomograms demonstrated the high accuracy of the conducted nomograms in predicting the survival time of patients in four cohorts (Figures 7I–L).

## Discussion

In the current study, through the mining of RNA-seq transcriptome data from diffuse glioma specimens, we conducted a comprehensive analysis to elucidate the relationship between glutamine metabolism and diffuse gliomas. To the best of our knowledge, glutamine metabolism is governed by numerous relevant enzymes and the driving force behind it, which involves gene expression and regulation. Hence, studying the expression pattern of GMRGs is a feasible approach to understanding the status of glutamine metabolism in glioma tumors. Unsupervised consensus clustering and prognostic gene signature screening were employed to classify diffuse gliomas into distinct groups, which helped us explore the influence of glutamine metabolism on diffuse gliomas lucidly.

Metabolic reprogramming is an eternal theme in cancer research. For a long time, it was thought that carcinogenesis and cancer progression required reprogramming their catabolic and anabolic metabolisms for energy supply and biomass synthesis (25). The most classic and well-known reprogrammed metabolic pathway in cancer is the Warburg effect, which discovers that cancer cells take up glucose and proceed to glycolysis regardless of oxygen availability (26, 27). Changes in glutamine metabolism are common metabolic alterations in cancer cells, ranking only after glucose metabolism (28). Glutamine accounts for ~4.7% of all amino acids in the human proteome. Beyond protein synthesis, glutamine also serves as a nitrogen donor, carbon donor, and amino acid precursor, and even participates in redox regulation and contributes to chromatin organization (29).

Glutamine metabolism has been studied in gliomas. Despite being a non-essential amino acid, “Glutamine addiction” extensively occurs in gliomas, particularly in GBMs (30). Elevated uptake of glutamine is accompanied by an increase in the malignancy of gliomas. In the study of Oizel et al. (31), results implied that mesenchymal GBM displayed the largest glutamine uptake compared to the brain tissue and other GBM subtypes. Other researchers also indicated that glutaminolysis dynamics during astrocytoma progression correlate with tumor aggressiveness (32). In addition, with the technique of high-performance liquid chromatography (HPLC), researchers found that glutamine required for the growth of GBM tumors was contributed partly by the circulation and was mainly either autonomously synthesized by GS-positive glioma cells or astrocytes (33).

Growing evidence disclosed that the expression and alteration status of genes related to glutamine metabolism not only reflect the actual biological process but also serve as the predictor of the prognosis of patients with cancer. An aberrant expression of GMRGs, such as GS, GLS, and glutamine transporter, was significantly associated with the prognosis in various cancers including breast cancer (34), hepatocellular carcinoma (35–37), ovarian cancer (38, 39), adrenal cortical neoplasms (40), lung squamous cell carcinoma (41), and intrahepatic cholangiocarcinoma (42). As for gliomas, high expression of GS and GLS was reported for both predicted poor prognoses (43, 44). In the prognostic model we conducted, a total of nine prognostic GMRGs (ASL, MTHFS, SLC7A7, GCLM, HAL, ADHFE1, FBLL1, SLC38A1, and OAT) were screened and included. The prognostic score based on the nine GMRGs exhibited powerful capability in predicting prognosis in patients with diffuse glioma.

Immune cells in TME, which are represented by macrophages, neutrophils, monocytes, lymphocytes, and NK cells, also underwent metabolic reprogramming along with the changes in cancer cells or stromal cells (45). In fact, metabolic reprogramming is also a hallmark of immune cell activation and is required for an antitumor immune response (9). Recent studies indicated that in the resting state and the activated state, there existed a significant difference in metabolic changes in immune cells (46, 47). Tumor cells could affect the immune cell function by competitively consuming nutrients in the TME, and metabolites from tumors that distribute in TME could also regulate immune cells (48). Here, we found that cluster 2 gliomas were characterized by significantly higher infiltration of M2 macrophages compared to cluster 1 gliomas, despite their IDH mutational status. This result was consistent with the result of a previous study which showed that glutamine synthesis was crucial in maintaining M2 polarization (49). Conversely, the lower plasma cell fraction in cluster 2 gliomas could be attributed to glutamine deprivation by the overpopulated tumor cell and M2 cells, which has been known to hinder the activation of B cells (50).

Based on the concept that glutamine plays an important role in the function of cancer cells and immune cells, anti-cancer strategies interfering with glutamine synthesis, transport, uptake, and other major biological processes have been developed in recent years. By targeting glutamine metabolism, the compound JHU083 was utilized, and the result indicated that glutamine blockade not only suppressed the growth of tumors but also remodeled the TME, which remarkably enhanced the efficacy of immunotherapy (51). Byun et al. (52) discovered the co-targeting of glutamine metabolism, and PD-L1 could significantly increase T cell-mediated cancer cell death. Clinical trials about the glutamine metabolism blockade in gliomas have been started in recent years. In the late stage of experimental GBM, a calorie-restricted ketogenic diet coordinated with glutamine antagonists could improve OS and relieve the symptoms of mouse models (53). Targeting glutaminase was also confirmed to be an effective means of inhibiting the growth of IDH1 mutant glioma cells (54).

There are still some inevitable limitations in the research. First, we only included cohorts of patients with glioma with clinicopathological data that were as complete as possible, and this might lead to selection bias. Second, in consideration that very few patients in these cohorts had received ICB therapy, the predictive ability of the therapeutic response for treating patients with glioma with ICBs was limited. Third, the current study only included primary diffuse gliomas, whether and how GMRGs affect recurrent gliomas is unknown. Fourth, actual metabolite detection from glioma specimens is needed to further verify the results from transcriptome data.

## Conclusion

In the current research, we conducted a comprehensive analysis based on transcriptome data to evaluate the relationship between glutamine metabolism and glioma biology. We found that the expression pattern of glutamine metabolism-related genes was closely associated with their IDH mutational status, but a significant distinction in tumor aggressiveness and TME profile persisted in both IDH-mutants and IDH-wildtype gliomas. A glutamine metabolism-related risk signature based on the expression of these genes could be utilized to predict the outcome of patients with glioma and integrated into a comprehensive prognostic model.

## Data availability statement

The datasets presented in this study can be found in online repositories. The name of the repository and accession number can be found below: National Genomics Data Center (NGDC), China National Center for Bioinformation (CNCB)/Beijing Institute of Genomics (BIG), Chinese Academy of Sciences (CAS), Genome Sequence Archive for Human (GSA-Human), https://ngdc.cncb.ac.cn/gsa-human/, HRA002839.

## Ethics statement

The studies involving human participants were reviewed and approved by the Ethical Committee of West China Hospital Sichuan University. The patients/participants provided their written informed consent to participate in this study.

## Author contributions

Conceptualization and writing-review and editing: XM and YL. Methodology: HF, SZ, and SC. Software and formal analysis: SZ, ZW, and YY. Resources: HF, SC, YY, ZW, and JL. Data curation: SZ. Result interpretation: HF and SZ. Writing-original draft: All authors. All authors contributed to the article and approved the submitted version.

## Supplementary material

Supplementary Figure 1 CIBERSORTx algorithm for evaluating TME immune cell infiltration of two clusters in CGGA325, CGGA693, and WCH cohorts, **(A–C)**; the ESTIMATE algorithm for evaluating TME immune characteristics of two clusters based on IDH mutational status in CGGA325 **(D, G, J)**; CGGA693 **(E, H, K)**; and WCH **(F, I, L)** cohorts. TME, tumor microenvironment; CGGA, Chinese Glioma Genome Atlas; WCH, West China Hospital; ESTIMATE, estimation of stromal and immune cells in malignant tumor tissues using expression data; IDH, isocitrate dehydrogenase.

Supplementary Figure 2 **(A–C)** ICP expression level of two clusters in CCGA325, CGGA693, and WCH cohorts. Tumor immunological phenotype analysis for classifying tumors as “cold” and “hot” based on IDH mutation status in CGGA325 **(D, G, J)**, CGGA693 **(E, H, K)**, and WCH **(F, I, L)** cohorts. ICP, immune checkpoint; TCGA, The Cancer Genome Atlas; CGGA, Chinese Glioma Genome Atlas; WCH, West China Hospital; IDH, isocitrate dehydrogenase.

Supplementary Figure 3 TIDE algorithm for predicting the therapeutic response based on IDH mutation status in CGGA325 **(A, D, G)**, CGGA693 **(B, E, H)**, and WCH **(C, F, I)** cohorts. TIDE, tumor immune dysfunction and exclusion; CGGA, Chinese Glioma Genome Atlas; WCH, West China Hospital; IDH, isocitrate dehydrogenase.

Supplementary Figure 4 Differences in clinicopathological variables between glutamine metabolism risk groups: age at diagnosis **(A)**, gender **(B)**, WHO grade **(C)**, IDH mutation status **(D)**, 1p19q codeletion status **(E)**, ATRX mutation status **(F)**, MGMT promoter status **(G)**, TERT promoter status **(H)**, histology **(I)**, and the waterfall plot for demonstrating the impact of each screened GMRG on clinic variables **(J)**. WHO, World Health Organization; IDH, isocitrate dehydrogenase; codel, codeletion; TERT, telomerase reverse transcription; MGMT, O6-methylguanine-DNA methyltransferase; ATRX, alpha thalassemia/mental retardation syndrome X-linked protein/gene; TCGA, The Cancer Genome Atlas; GMRG, glutamine metabolism-related gene.

Supplementary Figure 5 The optimal cutoff value of GMRS in CGGA 693 **(A)**, CGGA 325 **(B)**, and WCH **(C)** cohorts, respectively; K–M curves for assessing GMRS in CGGA 693 **(D)**, CGGA 325 **(E)**, and WCH **(F)** cohorts according to the optimal cutoff. CGGA, Chinese Glioma Genome Atlas; WCH, West China Hospital; GMRS, glutamine metabolism risk score.

Supplementary Table 1 Glutamine metabolism-related pathway acquired from MSigDB. MSigDB, molecular signature database.
