# Supplementary figures and images for: Glutamine metabolism-related genes predict prognosis and reshape tumor microenvironment immune characteristics in diffuse gliomas
Source: Front Neurol. 2023 Mar 10;14:1104738. doi: 10.3389/fneur.2023.1104738 (PMC10036600; doi:10.3389/fneur.2023.1104738)

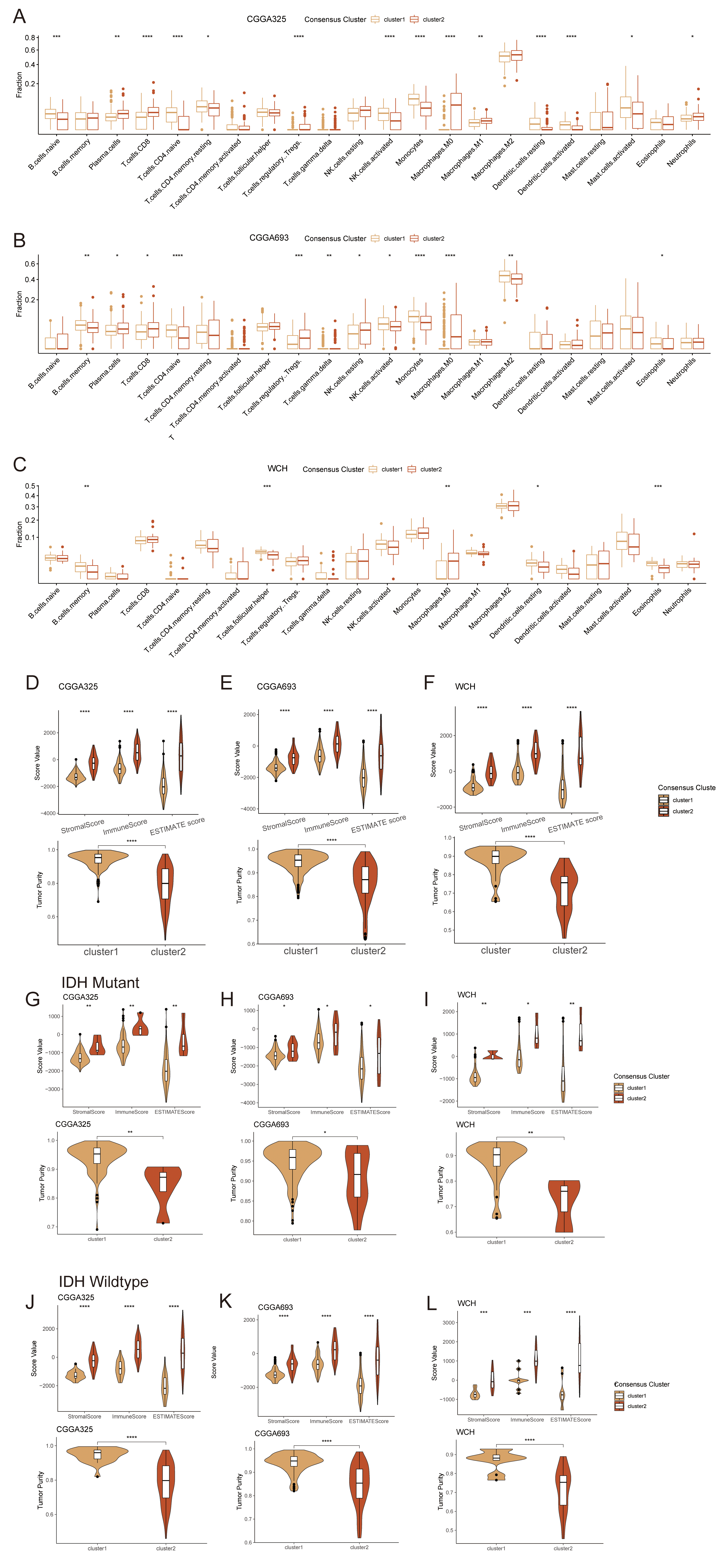

Supplement: Supplementary Figure 1 — CIBERSORTx algorithm for evaluating TME immune cell infiltration of two clusters in CGGA325, CGGA693, and WCH cohorts, (A–C); the ESTIMATE algorithm for evaluating TME immune characteristics of two clusters based on IDH mutational status in CGGA325 (D, G, J); CGGA693 (E, H, K); and WCH (F, I, L) cohorts. TME, tumor microenvironment; CGGA, Chinese Glioma Genome Atlas; WCH, West China Hospital; ESTIMATE, estimation of stromal and immune cells in malignant tumor tissues using expression data; IDH, isocitrate dehydrogenase. [file Image_1.TIF]

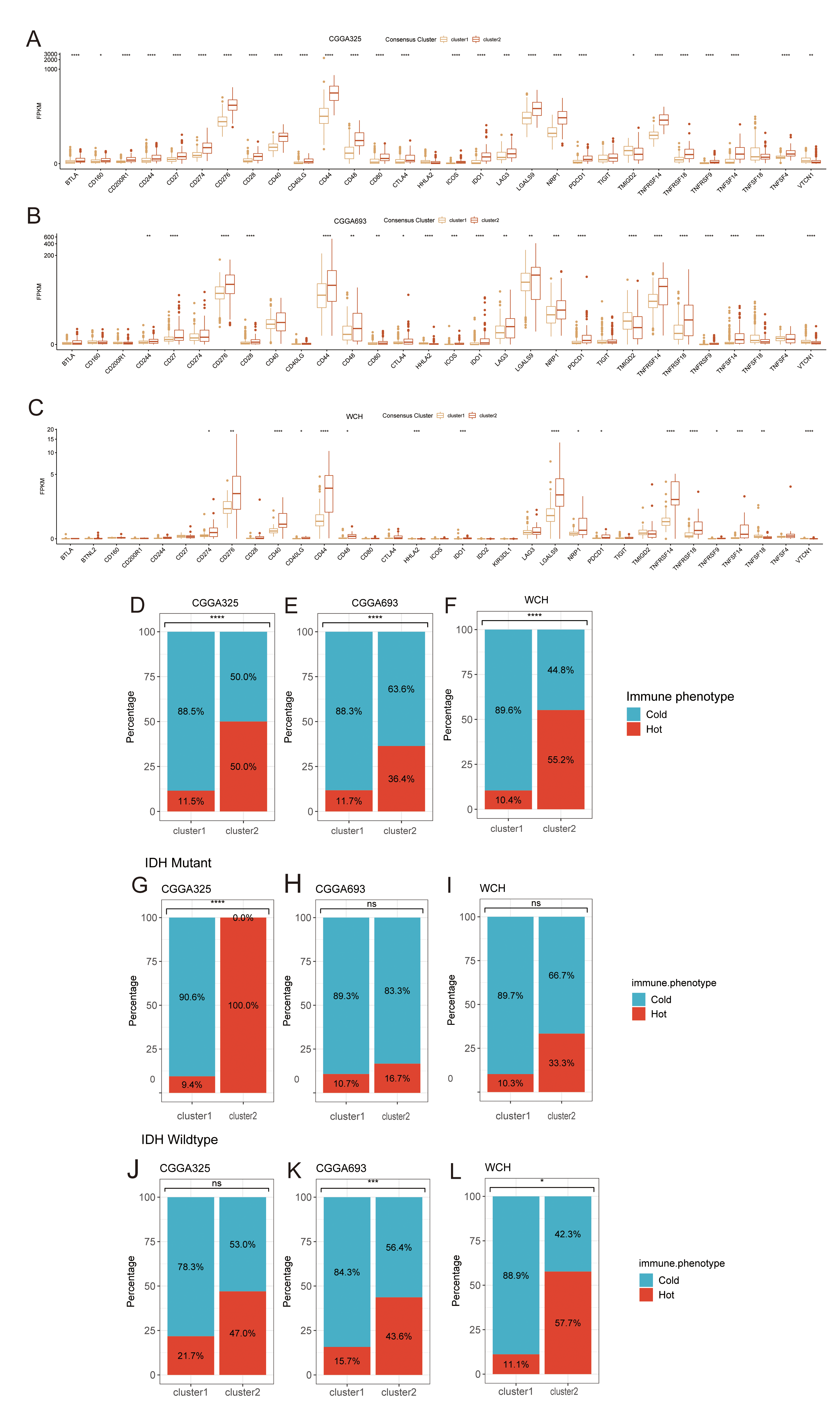

Supplement: Supplementary Figure 2 — (A–C) ICP expression level of two clusters in CCGA325, CGGA693, and WCH cohorts. Tumor immunological phenotype analysis for classifying tumors as “cold” and “hot” based on IDH mutation status in CGGA325 (D, G, J), CGGA693 (E, H, K), and WCH (F, I, L) cohorts. ICP, immune checkpoint; TCGA, The Cancer Genome Atlas; CGGA, Chinese Glioma Genome Atlas; WCH, West China Hospital; IDH, isocitrate dehydrogenase. [file Image_2.TIF]

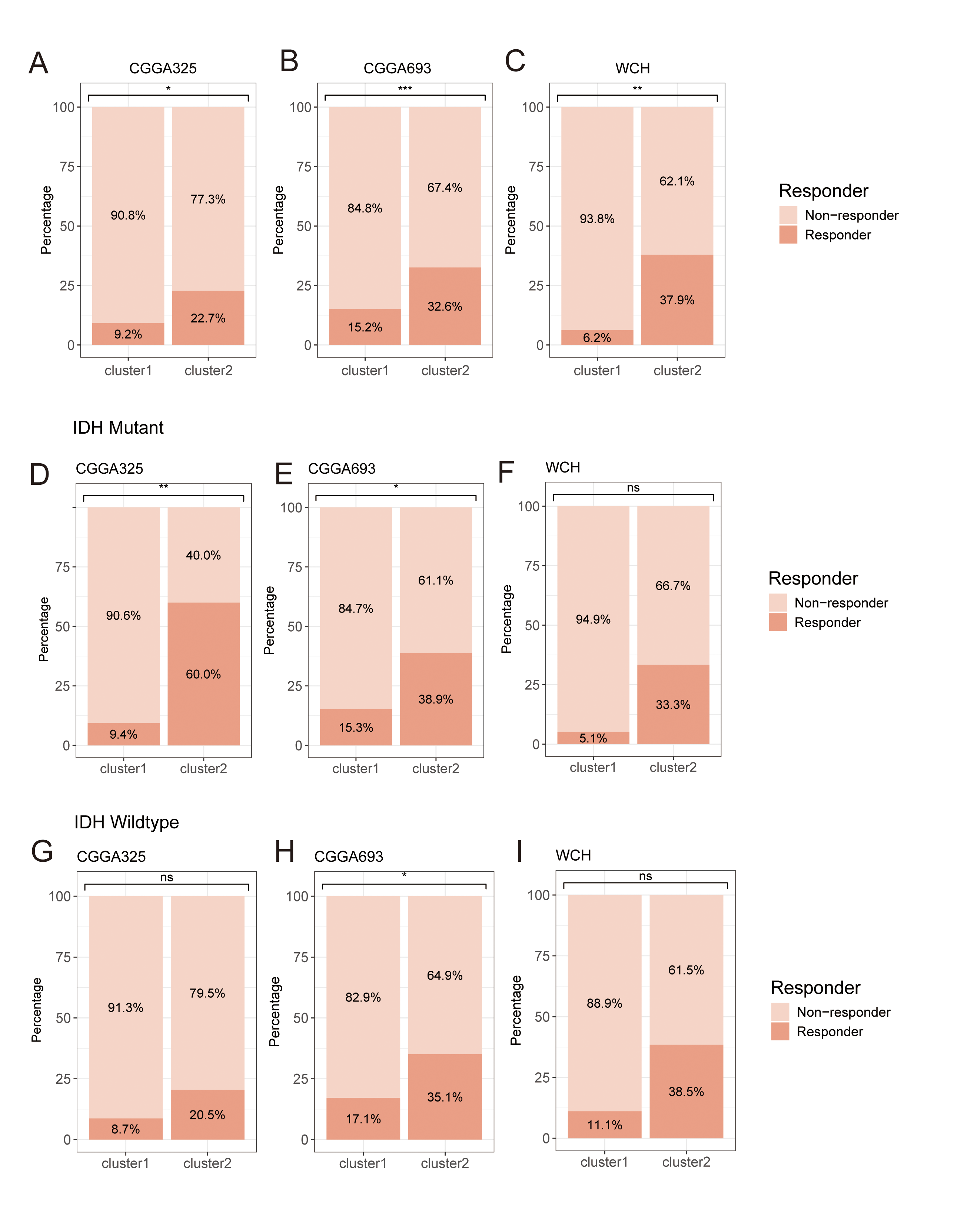

Supplement: Supplementary Figure 3 — TIDE algorithm for predicting the therapeutic response based on IDH mutation status in CGGA325 (A, D, G), CGGA693 (B, E, H), and WCH (C, F, I) cohorts. TIDE, tumor immune dysfunction and exclusion; CGGA, Chinese Glioma Genome Atlas; WCH, West China Hospital; IDH, isocitrate dehydrogenase. [file Image_3.TIF]

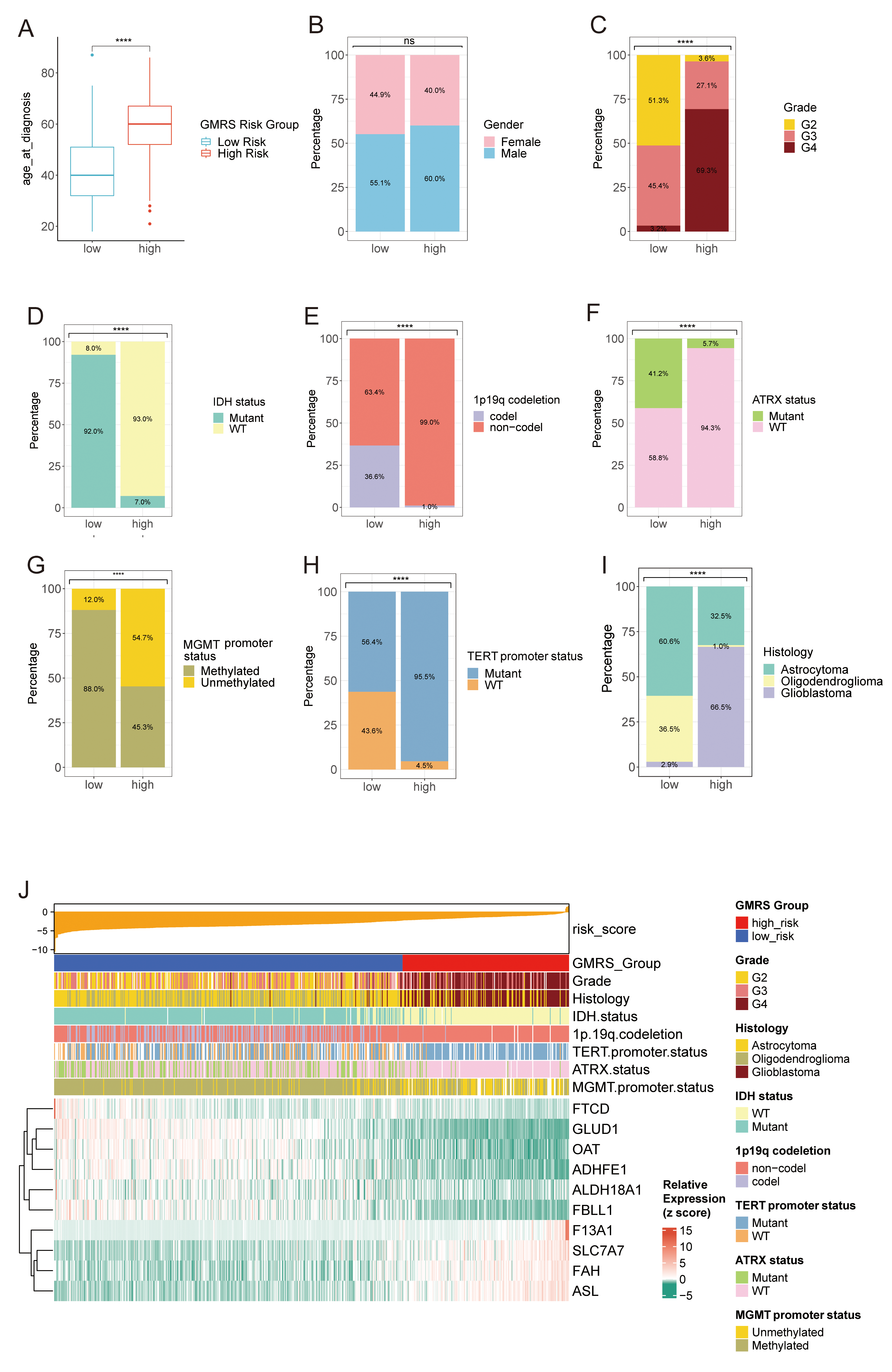

Supplement: Supplementary Figure 4 — Differences in clinicopathological variables between glutamine metabolism risk groups: age at diagnosis (A), gender (B), WHO grade (C), IDH mutation status (D), 1p19q codeletion status (E), ATRX mutation status (F), MGMT promoter status (G), TERT promoter status (H), histology (I), and the waterfall plot for demonstrating the impact of each screened GMRG on clinic variables (J). WHO, World Health Organization; IDH, isocitrate dehydrogenase; codel, codeletion; TERT, telomerase reverse transcription; MGMT, O6-methylguanine-DNA methyltransferase; ATRX, alpha thalassemia/mental retardation syndrome X-linked protein/gene; TCGA, The Cancer Genome Atlas; GMRG, glutamine metabolism-related gene. [file Image_4.TIF]

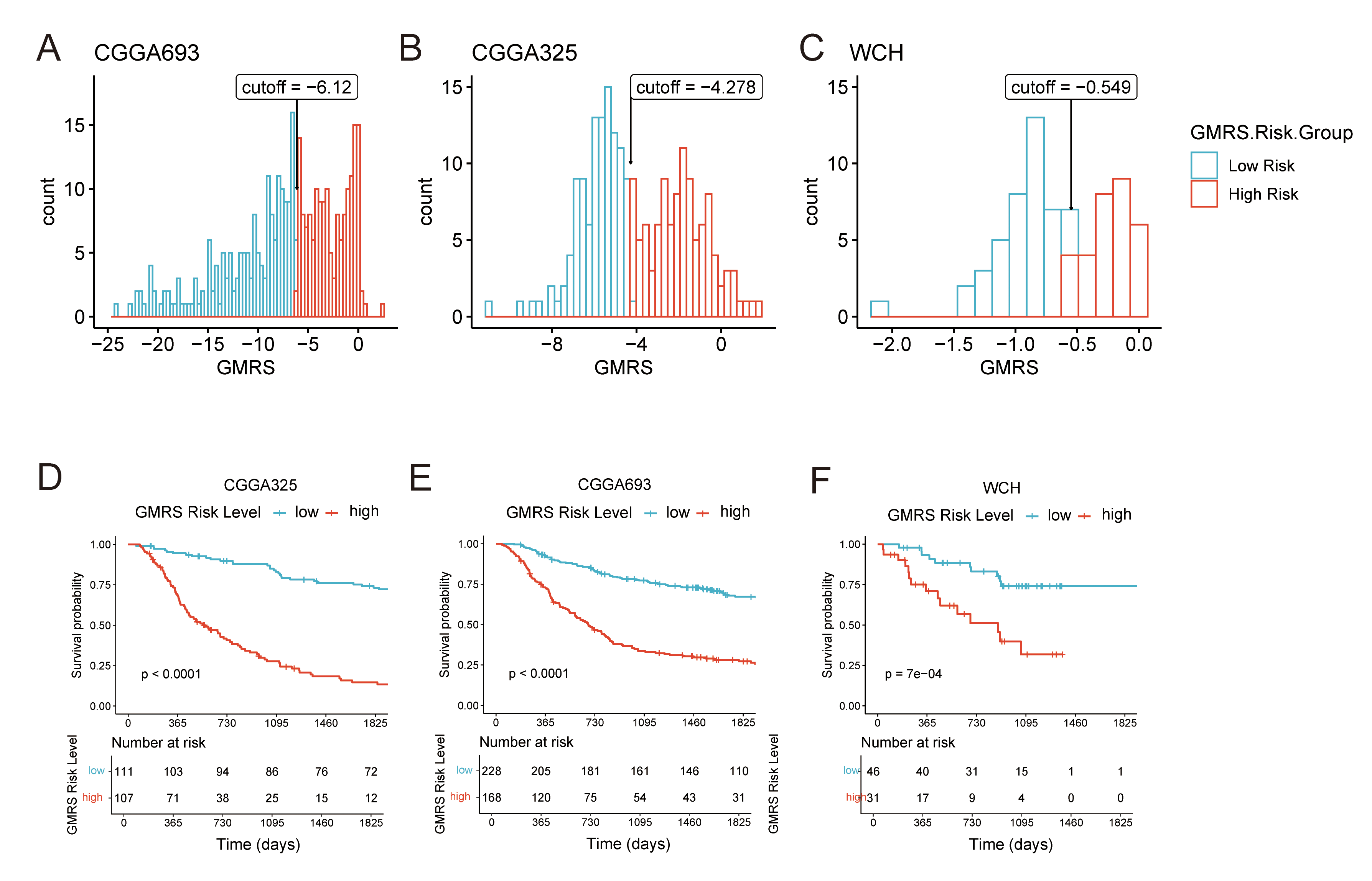

Supplement: Supplementary Figure 5 — The optimal cutoff value of GMRS in CGGA 693 (A), CGGA 325 (B), and WCH (C) cohorts, respectively; K–M curves for assessing GMRS in CGGA 693 (D), CGGA 325 (E), and WCH (F) cohorts according to the optimal cutoff. CGGA, Chinese Glioma Genome Atlas; WCH, West China Hospital; GMRS, glutamine metabolism risk score. [file Image_5.TIF]
